# Tympanic Bone: Small Neglected Bone with Extreme Anatomic Importance in Lateral Skull-Base Surgery

**DOI:** 10.1097/ONO.0000000000000085

**Published:** 2026-02-06

**Authors:** Golda Grinblat, Melcol Hailu Yilala, Wenlong Tang, Giuseppe Fancello, Mario Sanna

**Affiliations:** 1 Department of Otorhinolaryngology, Head and Neck Surgery, Hillel Yaffe Medical Center, Hadera, Israel; 2 Bruce Rappaport Faculty of Medicine, Technion-Israel Institute of Technology, Haifa, Israel; 3 Department of Otolaryngology-Head and Neck Surgery, Addis Ababa University, Ethiopia; 4 Department of Otology and Skull Base Surgery, Gruppo Otologico, Italy; 5 Department of Otolaryngology, Shenzhen Longgang Otolaryngology Hospital, and Shenzhen Otolaryngology Research Institute.

**Keywords:** Infratemporal fossa approaches, Skull base, Temporal bone, Tympanic bone, Tympanojugular paraganglioma

## Abstract

**Introduction:**

The tympanic bone (TB) is 1 of the 4 essential parts of the temporal bone, located on its lateral-inferior aspect. It plays a pivotal role in forming the bony part of the external auditory canal, facilitating the transmission of sound to the middle ear.

**Objective:**

The current study aims to underline the surgical importance of the TB and its role as a key structure in lateral skull-base surgery, particularly the infratemporal fossa approach type A and removal of the TB during the surgical management of tympanojugular paraganglioma (TJP).

**Materials and Methods:**

In this study, we examined 20 formalin-prepared temporal bones to highlight the significant anatomical importance of the tympanic portion and its association with the styloid process in TJP surgery. Furthermore, a comprehensive MEDLINE (PubMed) search was conducted using the MeSH term (tympanic bone).

**Results:**

The minimum, maximum, and average thicknesses of the TB were 9.3 mm, 12.2 mm, and 10.6 mm, respectively. Whereas the length of the styloid process ranged from 29 mm to 52 mm, with a mean measurement of 43 mm. Among the 272 TJP cases operated on at our center, there were (26%) C1, (49%) C2, (22%) C3, and (3%) C4 cases according to the modified Fisch classification. TB infiltration was evident in virtually all C3 and C4 cases, approximately 70% of C2 tumors, and about 40% of C1 tumors.

**Conclusion:**

Although the TB is rarely discussed explicitly as a distinct structure in the literature, it plays a crucial role in many otologic and lateral skull-base procedures. These surgeries frequently require the partial or complete removal of the TB to access the hypotympanum and the internal carotid artery. A meticulous study of high-resolution computed tomography scans, especially coronal sections, is essential for understanding the detailed anatomy of TB.

The tympanic bone (TB) is 1 of the 4 essential parts of the temporal bone, located on its lateral-inferior aspect. It plays a pivotal role in forming the bony part of the external auditory canal (EAC), facilitating the transmission of sound to the middle ear. Acting as a barrier, the TB separates the central part of the temporomandibular joint from the EAC. Moreover, the lower portion of the TB merges with the base of the styloid process, creating a substantial bony buttress that overlies the cervical internal carotid artery (ICA) (1). This anatomical configuration is depicted in Figure 1A–D (2), highlighting the TB’s structural and surgical significance.

**FIG. 1.**
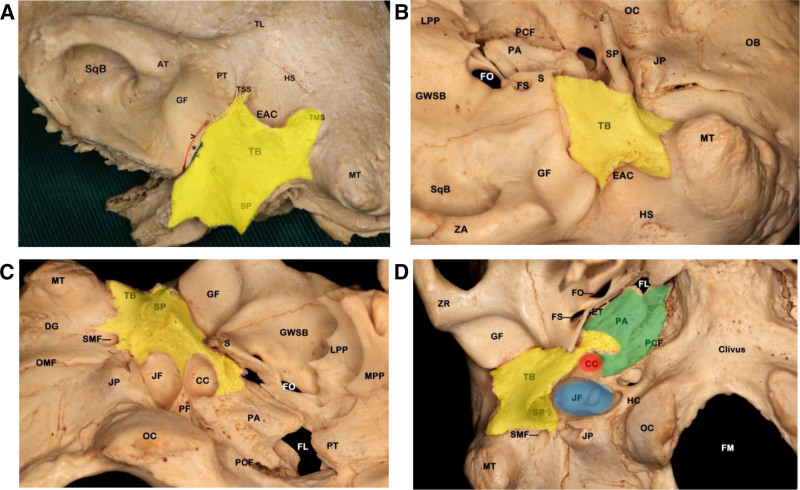
*A*, Inferiolateral view of the left temporal bone. There is a small piece of petrous bone between the squamous bone anteriorly and the tympanic bone posteriorly, forming the petrosquamous fissure and petrotympanic fissure, respectively. *B*, Inferolateral view of the right temporal bone, showing the adjacent relationship between the tympanic bone and surrounding bony structures. *C*, Inferomedial view of the left temporal bone, showing the adjacent relationship between the tympanic bone and surrounding bony structures. *D*, Inferior view of the right temporal bone, showing the adjacent relationship between the tympanic bone (yellow area), petrous apex (green area), carotid canal (red area), and jugular foramen (blue area). > indicates petrosquamous fissure; <, petrotympanic fissure; *, petrous bone; AT, anterior zygomatic tubercle; CC, carotid canal; DG, digastric groove; EAC, external auditory canal; FL, foramen lacerum; FM, foramen magnum; FO, foramen ovale; FS, foramen spinosum; GF, glenoid fossa; GWSB, greater wing of sphenoid bone; HC, hypoglossal canal; HS, Henle’s spine; JF, jugular foramen; JP, jugular process; LPP,lateral pterygoid plate; MPP, medial pterygoid plate; MT, mastoid tip; OB, occipital bone; OC, occipital condyle; OMF, occipitalmastoid suture; PA, petrous apex; PCF, petroclival fissure; PF, pyramid fossa; PT, posterior zygomatic tubercle; S, spine of sphenoid bone; SMF, stylomastoid foramen; SP, styloid process; SqB, squamosal part of the temporal bone; TB, tympanic bone; TL, temporal line; TMS, tympanomastoid suture; TSS, tympanosquamous suture; ZA, zygomatic arch.

According to Weinmann and Sicher, the development of the roof of the EAC occurs through extensive bone remodeling in the tympanic region (1). The C-shaped ring of the middle ear is identified as the immediate precursor to TB. This bone not only forms the floor and anterior wall of the auditory canal but is also integral to its overall structure and function. Typically, the auditory canal completes its development by the third year of a child’s life, although this timeline can vary among individuals (3). Despite the limited literature on the subject, there is considerable individual variation in the morphology of TB, highlighting the need for further research to better understand these differences (1).

As the smallest portion of the temporal bone, the TB is routinely drilled during many otologic and lateral skull-base approaches; however, its specific anatomical features are rarely discussed explicitly as a distinct structure in the literature. However, the complete removal of the TB easily reveals medially situated vital neurovascular structures, allowing complete tumor removal and less bleeding during tumor resection, and thus renders this bone great anatomical and surgical importance.

Furthermore, external and middle ear conditions that require widening of the EAC, such as canaloplasty and exposure of the hypotympanum, are performed after drilling the anterior and inferior portion of the TB. While partial or total TB removal is a crucial step in many otologic and lateral skull-base procedures, little is known about the appropriate TB thickness for a safe dissection (4).

In the present article, we would like to stress the anatomical importance of TB and its role as a “key structure” in lateral skull-base surgery, such as infratemporal fossa approach type A (IFTA-A) and subtotal petrosectomy, and in the removal of tumors of this site, in particular, tympanojugular paragangliomas (TJP) and tympanomastoid paragangliomas (TMP).

## AIM OF THE CURRENT STUDY

**FIG. 2.**
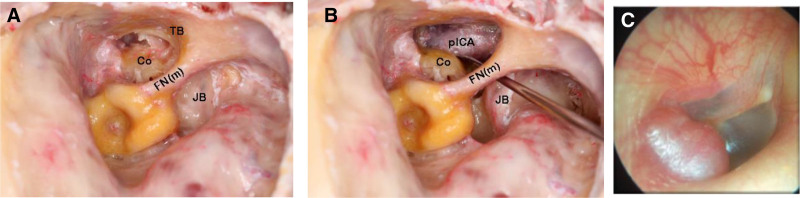
*A and B*, Relationship of the tympanic bone, facial nerve, and the jugular bulb: note that drilling the tympanic bone between the internal carotid artery and the mastoid segment of the facial nerve is a very important step to getting complete control of the jugular foramen area and the mastoid segment of the facial nerve and the vertical internal carotid artery. *C*, Characteristic otoscopic sign of retrotympanic mass with a rising sign appearance. Co indicates cochlea; FN(m), mastoid segment of the facial nerve; JB, jugular bulb; pICA, petrous segment of the internal carotid artery; TB, tympanic bone.

Why does the TB have such an important role? The TB plays an indispensable role, especially in the management of TJPs. The presence of this bone complicates the control of the ICA, making complete tumor removal challenging and increasing the risk of arterial damage during dissection. Consequently, the complete removal of the TB is essential for achieving thorough tumor excision and ensuring safe access to the ICA wall to perform meticulous dissection from the adventitia. Additionally, it is noteworthy that the jugular bulb (JB) is located superomedial to the inferior portion of the TB (Fig. 2A, B), which is frequently infiltrated by the lesion, resulting in the characteristic otoscopic appearance known as the “rising sun”(Fig. 2C) Thus, the significance of TB extends beyond its anatomical role, underscoring its crucial impact on surgical outcomes in TJP management (5).

**FIG. 3.**
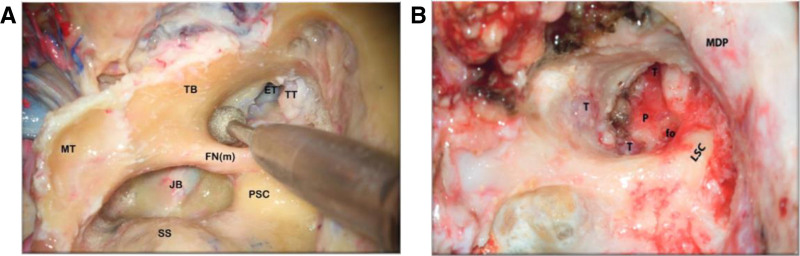
*A and B*, Drilling the tympanic bone between the internal carotid artery and the mastoid segment of the facial nerve is a very important step to getting complete control of the jugular foramen area and the mastoid segment of the facial nerve. ET indicates eustachian tube; FN(m), mastoid segment of facial nerve; fo, oval window; JB, jugular bulb; LSC, lateral semicircular canal; MDP, middle fossa dura plate; MT, mastoid tip; P, promontory; PSC, posterior semicircular canal; SS, sigmoid sinus; T, tumor; TB, tympanic bone; TT, tensor tympani muscle.

The laboratory research on 20 formalin-prepared temporal bones has been performed to demonstrate the tremendous anatomical importance of the tympanic portion with a styloid process in TJP surgery, conducting one to perform its complete removal. Hence, the consequence of complete TB elimination has a 3-fold meaning: 1) removal of tumor-infiltrated TB using a large diamond burr until the ICA is completely controlled, thus gaining complete control of nearby neurovascular structures, 2) removal of the styloid process, achieving control of the vertical portion of the ICA along with total tumor removal of infiltrated adventitia, hence reducing and avoiding incomplete tumor removal and recurrence, and 3) as a part of IFTA-A, drilling the retro-facial cells and a portion of TB between the ICA and the mastoid segment of the facial nerve (FN) facilitates its skeletonization while getting complete control of the jugular foramen (JF) area (Fig. 3A, B).

**FIG. 4.**
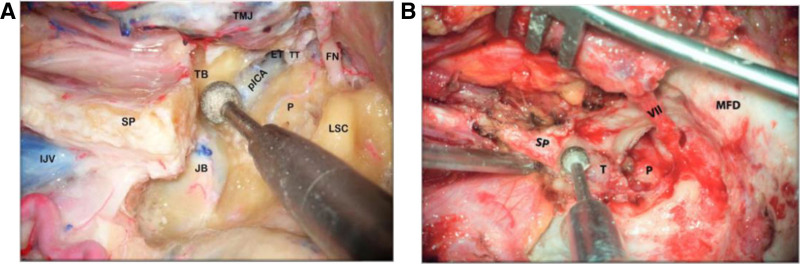
*A and B*, The tympanic bone is drilled out and the styloid process is removed to expose the petrous segment of the internal carotid artery. Note the relationship between the anterior wall of the jugular bulb and the tympanic bone. During the removal of the base of the styloid process, the utmost care has to be taken not to injure the cervical ICA lying immediately deep to it. ET indicates eustachian tube; FN, rerouted part of the facial nerve; IJV, internal jugular vein; JB, jugular bulb; LSC, lateral semicircular canal; MFD, middle fossa dura; P, promontory; pICA, petrous segment of internal carotid artery; SP, styloid process; T,tumor; TB, tympanic bone; TMJ, temporamandibular joint; TT, tensor tympani muscle; VII, rerouted part of the facial nerve.

Furthermore, when TB is drilled out, and the styloid process is removed to expose the petrous segment of ICA, one can create wider surgical access to perform safe tumor resection, reducing the chance of injury to the lower cranial nerves (LCNs), which are situated medial to the JB. Note the relationship between the anterior wall of the JB, the petrous ICA, and the TB (Fig. 4A, B). During the removal of the base of the styloid process, utmost care has to be taken not to injure the cervical ICA, which lies immediately deep into it.

**FIG. 5.**
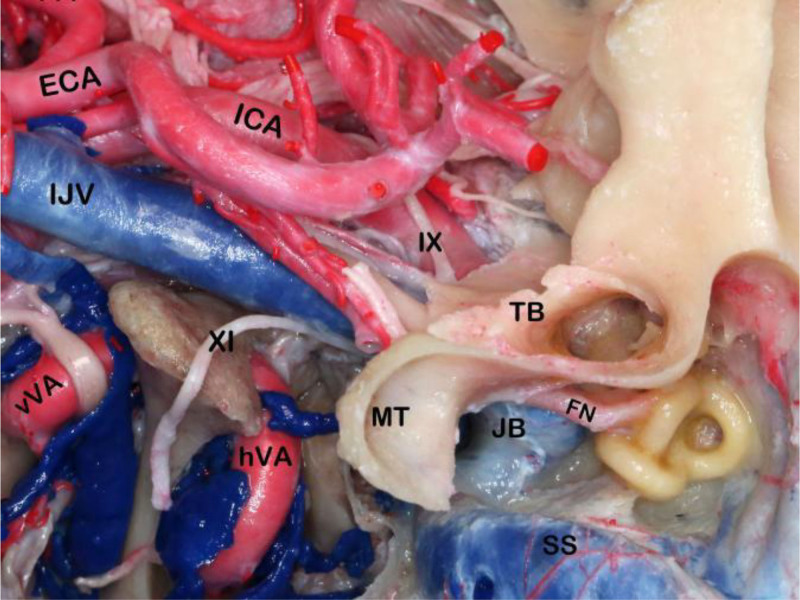
The relation of the tympanic bone with the MT, JB, FN, and more medially situated structures of the skull base, such as the ICA and lower cranial nerves. ECA indicates external carotid artery; FN, facial nerve; hVA, horizontal vertebral artery; ICA, internal carotid artery; IJV, internal jugular vein; IX, glossopharyngeal nerve; JB, jugular bulb; MT, mastoid tip; SS, sigmoid sinus; TB, tympanic bone; vVA, vertical vertebral artery; XI, spinal accessory nerve.

Hence, this technique of TB removal has been part of routine surgical procedures during the removal of TJP in our center for over three decades. Finally, in the literature, TB has not been mentioned in all the papers regarding TJP except in the book of Hugo Fisch, which briefly states the importance of removal of this bone during the IFTA and transotic (TO) approaches (Fig. 5) (6).

The current study aims to underline the surgical importance of the TB and its role as a key structure in lateral skull-base surgery, particularly IFTA-A and removal of the TB during the surgical management of TJP.

## MATERIALS AND METHODS

For the present study, a comprehensive MEDLINE (PubMed) search was conducted using the MeSH term (tympanic bone). The search yielded only 3 articles that specifically addressed the surgical and radiologic aspects of TB. The majority of the other cited articles briefly touched upon the significance of partially removing TB during middle ear procedures such as canaloplasty (2–4), and only 1 article (5) had a very short section written regarding the importance of TB in the lateral skull-base procedures.

In this study, we examined 20 formalin-prepared temporal bones to highlight the significant anatomical importance of the tympanic portion of the temporal bone and its association with the styloid process in TJP surgery. All dissected temporal bones were from adults, and the measurement was performed using a caliper. The thickness of the TB was measured from the mid-point of the 2 sutures: tympanosquamous and tympanomastoid suture lines, to the most inferior point of this bone. Whereas the length of the styloid process is measured from its base at the temporal bone to its distal tip.

By meticulously analyzing these specimens, we were able to demonstrate how the tympanic portion, when considered in conjunction with the styloid process, plays a crucial role in surgical procedures. The analysis included the morphology of the TB and its relationship with the JB, ICA, and FN. Our findings underscore the necessity of thorough removal of the TB to ensure optimal exposure and access to critical neurovascular structures during the surgical management of TJP, particularly the vertical portion of the ICA.

Additionally, we assessed the involvement of TB in patients who underwent surgery for TJPs over the past 30 years by analyzing intraoperative notes documented immediately after surgery. We studied the likelihood of involvement of TB for each class TJP (modified Fisch classification) (7).

## RESULTS

### Cadaveric Measurements

The minimum, maximum, and average thickness of the TB were 9.3 mm, 12.2 mm, and 10.6 mm, respectively. Whereas the length of the styloid process measured from its base at the temporal bone to its distal tip ranged from 29 mm to 52 mm, with a mean measurement of 43 mm (Table 1).

**TABLE 1.** Measurements of cadaveric dissection

Measurements	Minimum(mm)	Maximum(mm)	Average(mm)
Thickness of the TB	9.3	12.2	10.6
Length of the SP	29	52	43

TB indicates tympanic bone; SP, styloid process.

After drilling of the body of the TB in its whole thickness was accomplished, it was clear that just immediately medially to it, JB was discovered, which is within 1–2 mm distance medially. Moreover, the mastoid portion of FN was always seen in continuation with the drilled medio-inferoposterior border of the TB. Finally, the vertical portion of the ICA could be measured after complete TB body removal, providing maximal access to a styloid process that was always in very intimate relation to the vertical portion of the ICA that was practically attached to it. However, the distance from the FN, JB, and ICA was very difficult to measure on the formalin specimens before the drilling.

### Intraoperative Findings

Among the 272 TJP cases operated on at our center, there were (26%) C1, (49%) C2, (22%) C3, and (3%) C4 cases according to the modified Fisch classification.

TB infiltration was evident in virtually all C3 and C4 cases, approximately 70% of C2 tumors, and about 40% of C1 tumors (Table 2). This data underscores the frequent involvement of TB in TJP and highlights its significance in surgical planning and management.

**TABLE 2.** *Percent and extent of tympanic bone involvement for each class of TJP (modified Fisch*)

TJPs (modified Fisch class)	Involvement of TB (%)
C1	40%
C2	70%
C3	100%
C4	100%

TB indicates tympanic bone; TJP, tympanojugular paraganglioma.

## DISCUSSION

While the removal of the TB is a crucial step in many otologic procedures, there is limited understanding regarding the thickness of the TB in areas suitable for safe dissection. Extensive studies have thoroughly examined the posterior portion of the TB, highlighting its relationship with the tympanic annulus, the EAC, and the FN. However, the anterior-inferior portion of the TB, which requires substantial removal to access the hypotympanum, JF region, and the vertical portion of the ICA, has not been thoroughly investigated. This area is particularly critical because generous removal is necessary to gain adequate exposure for surgical procedures. The lack of detailed anatomical information regarding the thickness and structural characteristics of TB in this region poses challenges for surgeons. This gap emphasizes the need for further research to ensure safe and effective dissection while minimizing risks to vital structures such as the ICA and surrounding neurovascular elements. Understanding these nuances is paramount to improving surgical outcomes and reducing complications in otologic and lateral skull-base procedures (4,8).

**FIG. 6.**
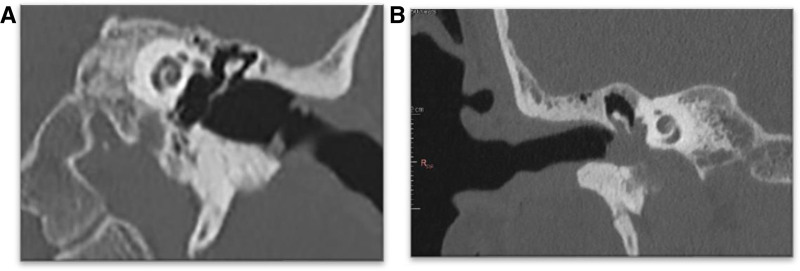
*A and B*, Coronal HRCT of normal and tumor-infiltrated TB: note the relationship of the tympanic bone with the middle ear cavity, cochlea, and hypotympanum. HRCT indicates high-resolution computed tomography; TB, tympanic bone.

From a radiologic perspective, high-resolution coronal CT scanning offers invaluable insights into the anatomical and pathological nuances of TB and its surrounding structures. This imaging modality provides detailed information on the thickness of the TB, the extent of its involvement by lesions affecting the JF region, particularly TJPs, and its proximity to crucial vascular systems (Fig. 6A, B). The coronal sections of high-resolution computed tomography (HRCT) scans are especially critical as they enable precise assessment of the TB’s structural integrity and the degree of infiltration by pathological processes. This thorough evaluation is essential for determining the full extent of the lesion, guiding surgical planning, and predicting potential complications (4).

In the context of TJP, where TB infiltration is almost always present, HRCT scans play a pivotal role in preoperative planning and intraoperative navigation. Understanding the detailed anatomy and pathology of TB helps surgeons anticipate and mitigate risks, such as damage to the ICA, LCNs, and other vital neurovascular structures. Consequently, the comprehensive study of HRCT scans is paramount in ensuring successful surgical outcomes and improving patient prognosis as a result of the complete removal of this important bone.

Because it provides access to the tympanic membrane and aids in the formation of a well-formed and calibrated mastoid cavity, drilling the TB is crucial in the treatment of problems affecting the external and middle ear cavities. Hence, the inferior and anterior walls serve as the canaloplasty’s initial starting points and excellent exposure for the resection of middle ear tumors such as TMPs. Consequently, the removal of class B3 TMP is provided by exposure of the hypotympanum and canaloplasty with a large excavation of the inferior TB using a subtotal petrosectomy approach (2).

Drilling the TB is a crucial step in treating conditions affecting the external and middle ear cavities. This procedure provides essential access to the tympanic membrane and contributes to the formation of a well-formed and calibrated mastoid cavity. The inferior and anterior walls of the TB serve as the initial starting points for canaloplasty, ensuring optimal exposure for the resection of middle ear tumors, such as TMPs. Specifically, the removal of class B3 TMPs is facilitated by exposing the hypotympanum and performing canaloplasty, which involves substantial excavation of the inferior TB using a subtotal petrosectomy approach (9). This comprehensive surgical technique enables the thorough removal of the tumor while preserving critical anatomical structures, thereby enhancing surgical outcomes and minimizing the risk of complications.

It is an undeniable fact that the inferior portion of the TB is the thickest. Removing this section provides maximal exposure to the underlying regions, particularly the hypotympanum and its crucial neurovascular relationships. This extensive exposure is essential for effective surgical access and visibility, allowing for the identification and preservation of vital structures. In particular, this approach can reveal critical structures such as the ICA and the JB. Given the involvement of the TB in TJPs, generous anteroinferior drilling of the TB is a critical initial step in identifying these structures during lateral skull-base approaches. The meticulous removal of the inferior TB allows surgeons to navigate and operate within this complex anatomical area with greater precision. Furthermore, the detailed anatomical understanding of the TB’s thickness and its relationship with surrounding structures is paramount for the success of surgical procedures. This thorough exposure, facilitated by drilling, not only aids in the complete removal of tumors but also ensures that surgeons can perform their tasks with minimal complications. By minimizing the risk of damaging vital neurovascular structures, such as the ICA, JB, and FN, this approach enhances the safety and efficacy of surgical procedures in this complex anatomical area. The precise identification and preservation of these crucial elements are critical for reducing intraoperative risks and improving patient outcomes (6,10).

In lateral skull-base surgeries, such as subtotal petrosectomy, transotic approach, and most importantly, the IFTA-A, the removal of the TB is an essential step. This is particularly crucial in surgical operations involving the rerouting of the FN, exposure of the hypotympanum, and JF, as well as the control of the vertical segment of the ICA. The meticulous removal of the TB provides the necessary access and visibility to these vital structures, ensuring effective surgical intervention and minimizing the risk of complications. This thorough approach is critical for achieving successful outcomes in complex lateral skull-base procedures (6,10,11).

**FIG. 7.**
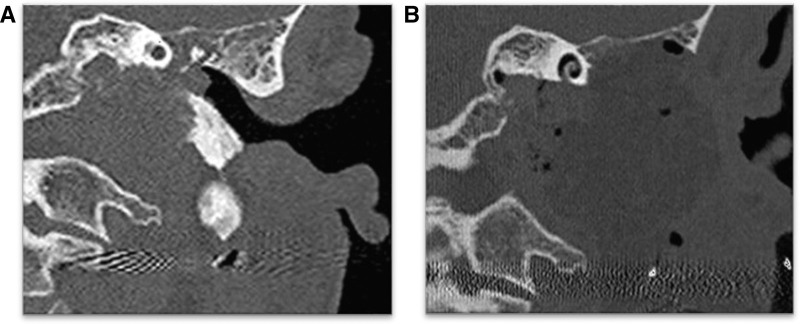
*A and B*, HRCT-Coronal section demonstrating the pre- and postoperative coronal view of a tympanic bone infiltrated by TJP and removed during IFTA-A. HRCT indicates high-resolution computed tomography; IFTA-A, infratemporal fossa approach type A; TB, tympanic bone; TJP, tympanojugular paraganglioma.

Removing the TB in front of the stylomastoid foramen allows for increased anterior exposure, significantly improving the surgeon’s view of the anterolateral portion of the JF region. This is particularly important for accessing tumors that extend towards the vertical segment of the intrapetrous ICA, which cannot be effectively reached using the Fallopian bridge technique. The thorough removal of the TB enhances the visualization and manipulation of neurovascular structures, facilitating complete tumor excision with minimal bleeding and minimizing the risk of injury to the LCN during surgery (Fig. 7A, B).

By providing clearer access and better control over critical anatomical structures, this approach plays a vital role in the management of TJPs. Therefore, the TB is instrumental in surgical planning and execution, ensuring that surgeons can achieve successful outcomes with greater precision and safety (5,12).

## LIMITATIONS OF THE CURRENT STUDY

1. Limited literature: The study highlights the scarcity of published literature specifically addressing the anatomical and surgical dimensions of the TB. This limited body of work may affect the comprehensiveness of the study’s conclusions and comparisons.
2. Sample size: The study utilized 20 formalin-prepared temporal bones. While this sample size allows for detailed examination, it may not be representative of the broader population, potentially limiting the generalizability of the findings.
3. Intraoperative note analysis: The assessment of TB involvement in patients over the past 20 years was based on intraoperative notes. Although these notes provide valuable insights, they may be subject to variations in documentation quality and detail, which could affect the accuracy of the data collected.
4. Individual variation: The study acknowledges considerable individual variation in TB morphology, which may impact the consistency of findings. Further research is needed to better understand these differences and their implications for surgical practice.
5. Anatomical and surgical emphasis: The study primarily focuses on the anatomical and surgical importance of TB. It may benefit from a broader perspective that includes the functional and clinical implications of TB involvement in various otologic and lateral skull-base procedures.

## CONCLUSIONS

Although the TB is rarely discussed explicitly as a distinct structure in the literature, it plays a crucial role in many otologic and lateral skull-base procedures. These surgeries frequently require the partial or complete removal of the TB to access the hypotympanum and the ICA. A meticulous study of HRCT scans, especially coronal sections, is essential for understanding the detailed anatomy of TB. One of the limitations of our study is the paucity of extensive research on TB, which restricted our ability to compare our findings with existing literature.

## FUNDING SOURCES

This research received funding from the Mario Sanna Foundation.

## CONFLICTS OF INTEREST

None declared.

## DATA AVAILABILITY STATEMENT

The data presented in this study are available in this article.

## References

[1] Eckerdal O Ahlqvist J Alehagen U Wing K. Length dimensions and morphologic variations of the external bony auditory canal: A radiographic and histologic investigation. Dentomaxillofac Radiol. 1978;7:43–50. 291554 10.1259/dmfr.1978.0006

[2] Atlanoğlu S Topuz MF. Surgical importance of the tympanic bone: multidetector computed tomography findings. J Korean Assoc Oral Maxillofac Surg. 2022;48:149–154. 35770355 10.5125/jkaoms.2022.48.3.149 PMC9247452

[3] Eckerdal O Ahlqvist J. External bony auditory canal and the tympanic bone. Acta Radiol Diagn. 1980;21:425–431. 10.1177/028418518002100314 7435227

[4] Rodrigues S Fagan P Doust B Moffat K. A radiologic study of the tympanic bone: anatomy and surgery. Otol Neurotol. 2003;24:796–799. 14501458 10.1097/00129492-200309000-00017

[5] Keles B Semaan MT Fayad JN. The medial wall of the jugular foramen: a temporal bone anatomic study. Otolaryngol Head Neck Surg. 2009;141:401–407. 19716021 10.1016/j.otohns.2009.05.030

[6] Ugo F Mattox D. Microsurgery of the Skull Base. Thieme Publishers Series; 1988. 163–244 p.

[7] Sivalingam S Konishi M Shin SH Lope Ahmed RA Piazza P Sanna M. Surgical management of tympanojugular paragangliomas with intradural extension, with a proposed revision of the Fisch classification. Audiol Neurootol. 2012;17:243–255. 22584244 10.1159/000338418

[8] Mario S Saleh E Khrais T. Atlas of Microsurgery of the Lateral Skull Base;2008. p. 80–129.

[9] Yilala MH Fancello G Fancello V Lauda L Sanna M. Long-term surgical outcome of class A and B tympanomastoid paragangliomas. Cancers (Basel). 2024;16:1466. 38672548 10.3390/cancers16081466 PMC11048109

[10] Mario S. Microsurgery of skull base Paragangliomas. Thieme Publishers Series; 227–228 p.

[11] Prasad SC Roustan V Piras G Caruso A Lauda L Sanna M. Subtotal petrosectomy: Surgical technique, indications, outcomes, and comprehensive review of literature. Laryngoscope. 2017;127:2833–2842. 28349533 10.1002/lary.26533

[12] Prasad SC Mimoune HA Khardaly M Piazza P Russo A Sanna M. Strategies and long-term outcomes in the surgical management of tympanojugular paragangliomas. Head Neck. 2016;38:871–885. 26343411 10.1002/hed.24177

